# A Case of Stevens–Johnson Syndrome Induced by Selpercatinib

**DOI:** 10.1111/1346-8138.17749

**Published:** 2025-04-16

**Authors:** Yuki Tone, Mami Matsui, Aeri Park, Saki Otani, Mizuki Shiba, Fumisa Okano, Iwao Sugitani, Naoko Kanda, Hidehisa Saeki, Toshihiko Hoashi

**Affiliations:** ^1^ Department of Dermatology Nippon Medical School Bunkyo‐Ku Tokyo Japan; ^2^ Department of Endocrine Surgery Nippon Medical School Bunkyo‐Ku Tokyo Japan; ^3^ Department of Dermatology Nippon Medical School, Chiba Hokusoh Hospital Inzai Chiba Japan

**Keywords:** adverse event, medullary thyroid carcinoma, selpercatinib, Stevens–Johnson syndrome, toxic epidermal necrolysis

## Abstract

Stevens–Johnson syndrome (SJS) and toxic epidermal necrolysis are fatal adverse skin reactions characterized by high fever, epidermal detachment, and mucositis. Selpercatinib is a highly selective inhibitor of tyrosine kinase, rearranged during transfection (RET), and is the first targeted therapy for solid tumors with *RET* gene alteration. The main adverse events of selpercatinib include hypertension, liver dysfunction, diarrhea, and QT prolongation on electrocardiograms. We present the case of 50‐year‐old male with medullary thyroid carcinoma who was treated with selpercatinib. On the 12th day of the treatment, he developed widespread erythema, which resulted in a referral to our department. Physical examination revealed disseminated erythematous macules, conjunctival congestion, and bloody crusting of the lips, and histopathological examination showed single‐cell necrosis in epidermis. Based on these findings, he was diagnosed with SJS. After the discontinuation of selpercatinib and systemic administration of corticosteroids, his symptoms were improved. The patient showed positivity for selpercatinib in drug lymphocyte stimulation test, suggesting the diagnosis of SJS induced by selpercatinib. To the best of our knowledge, this is the first report of SJS caused by selpercatinib. We herein present and discuss this case to raise awareness.

## 1 Introduction

Rearranged during transfection (RET) is a single‐pass receptor tyrosine kinase, and alterations in *RET* gene are involved in the pathogenesis of several cancer types [1, 2]. By blocking RET signaling pathways, the selective RET inhibitors can effectively inhibit tumor growth, reduce the spread of cancer, and improve patients' outcomes. This innovative approach has revolutionized the treatment of *RET*‐altered cancers, offering new hope and improved outcomes on patients with *RET*‐altered cancer. Selpercatinib, which is a tyrosine kinase inhibitor (TKI) and highly selectively inhibits RET, is considered as the first RET inhibitor for adults with metastatic or locally advanced solid tumors with *RET* gene alteration [3].

Stevens–Johnson syndrome (SJS) and toxic epidermal necrolysis (TEN) are rare, acute, and life‐threatening T cell‐mediated mucocutaneous diseases characterized by extensive necrosis and detachment of the epidermis, and are associated with a high mortality rate [4, 5, 6]. SJS/TEN usually occurs after prodromal illness resembling an infection of upper respiratory tracts for several days. The prodromal illness includes fever (body temperature > 39°C), sore throat, rhinorrhea, cough, conjunctivitis and arthralgia. Recently, SJS/TEN is categorized into two distinct types based on the degree of skin involvement; skin detachment of < 10% of total body surface area is categorized as SJS, and ≥ 10% as TEN [7], with reported mortality rate 1%–10%, and 20%–40%, respectively [8]. We herein report a case of SJS induced by selpercatinib.

## 2 Case Report

**FIGURE 1:**
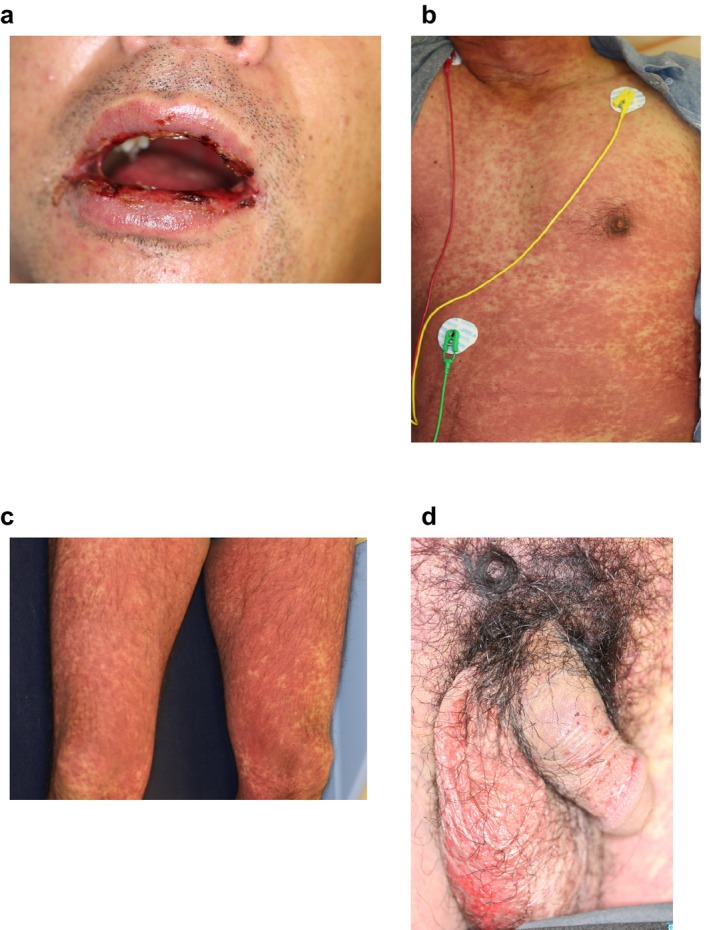
Clinical features at the time of admission. (a) Crusts on the lips and hemorrhagic erosions. (b, c) Atypical target lesions and palpable erythema with a tendency to confluence on the trunk and on the thighs. (d) Erosions on the scrotum and on the penile mucosa.

**FIGURE 2:**
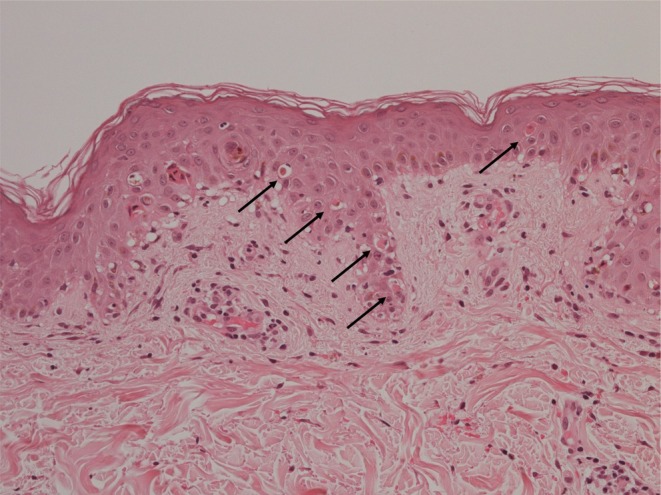
Histopathological examination. Biopsy from the erythematous macules on the abdomen showed single‐cell necrosis of epidermal cells (arrows), spongiosis in the epidermis, interface dermatitis, and inflammatory cell infiltration extending from the dermis into the epidermis (hematoxylin–eosin stain, original magnification ×200).

A 50‐year‐old male had medullary thyroid carcinoma with multiple pulmonary metastases. *RET* gene abnormality of C618R was obtained by the Oncomine CDx system using specimens of surgically resected medullary thyroid carcinoma. Selpercatinib was started at dose of 320 mg/day. Azilsartan was also started at the same time. Immunocheckpoint inhibitors were not administered. On the 12th day of treatment, he developed widespread erythema, leading to referral to our department. At the initial examination, we observed confluent and palpable atypical target lesions mainly on the trunk and thigh, bloody crusts on the lips, and erosions on the penile mucosa (Figure 1). He complained itching over face and trunk, and pain of oral mucosa. His body temperature was 39.9°C. Laboratory tests revealed alanine aminotransferase (ALT) of 46 IU/L, aspartate aminotransferase (AST) of 51 IU/L, white blood cell count of 6100/μL (neutrophils 82%, lymphocytes 3%, eosinophils 5.5%, and atypical lymphocytes < 4%), creatinine of 1.15 mg/dL, and C‐reactive protein of 34.56 mg/dL, indicating mild liver and renal dysfunction as well as an elevated inflammatory response. Histopathological examination showed multiple single‐cell necrosis of epidermal cells, strong spongiotic changes in the epidermis, and inflammatory cell infiltration extending from the dermis into the epidermis, consistent with the findings of SJS/TEN (Figure 2). Since the total skin detachment area was < 10%, the patient was diagnosed with SJS [5, 9]. To identify the causative drug, a drug‐induced lymphocyte stimulation test (DLST) was performed. The DLST showed significantly elevated stimulation index (SI; normal range, < 180%) for selpercatinib (SI, 130% on the 23th day, and 260% on the 79th day), suggesting selpercatinib as the causative drug for SJS. We discontinued selpercatinib treatment on the 16th day. Methylprednisolone pulse therapy (intravenous 1000 mg for three consecutive days) was administered, followed by oral prednisolone (1 mg/kg/day) and gradual dose tapering with no recurrence. Currently, the patient has sensory disturbances on the tongue but otherwise improved.

## 3 Discussion

SJS/TEN, with an incidence of 1.2–7.4/10,00000 among adults [5], is a rare and painful disease clinically characterized by epidermal exfoliation and systemic symptoms. The pathogenesis of SJS/TEN involves antigenic moiety/metabolite, peptide‐induced T cell activation, soluble Fas ligand, perforin/granzyme B, tumor necrosis factor‐alpha, nitric oxide, and granulysin [7, 10]. Such complicated pathogenesis makes it hard to standardize the therapeutic strategy for SJS/TEN. The effective treatments for SJS/TEN include early diagnosis, immediate withdrawal of suspicious allergenic drugs, symptomatic and supportive treatment. As for the treatment, corticosteroids are still the mainstay but bear a high risk of bacterial infections or sepsis, and do not seem to be superior to supportive care alone [7]. The sensitivity analysis based on multiple studies shows a trend towards a beneficial effect of intravenous immunoglobulin in the aspects of mortality. Cyclosporine A seems to be a promising therapy though it is unsuitable for patients with renal failure and/or immune deficiency [5]. Intensive skin care is crucially needed for the repair of skin barrier. Meanwhile, efforts should be done to control infection, including closely monitoring infectious signs and giving timely anti‐infectious treatments.

Though the development and increasing usage of selpercatinib improved survival of patients with *RET*‐altered cancer, it is crucial to be aware of its adverse events (AEs) [11]. The most common AEs (> 25%) were edema, diarrhea, fatigue, dry mouth, hypertension, abdominal pain, constipation, rash, nausea, headache, and QT prolongation on electrocardiograms [11]. The most common (≥ 5%) grade 3 or 4 laboratory abnormalities were lymphopenia, increased ALT or AST, decreased sodium or calcium. Some selpercatinib‐associated serious AEs leading to dose reduction or treatment interruption have also been reported; hypertension, elevated ALT or AST, and severe gastrointestinal toxicities characterized by small bowel edema and lymphocytic duodenitis [3]. Hypersensitivity occurred in 4.3% (grade 3 in 1.6%) of patients receiving selpercatinib. Signs and symptoms of hypersensitivity included fever, rash, and arthralgias or myalgias with concurrent decreased platelets or elevated transaminases. The median time to onset was 1.7 weeks (ranging 6 days to 1.5 years). Most patients were able to reinitiate selpercatinib at a lower dose in combination with corticosteroids, as described in the product label [11].

Selpercatinib is an anti‐cancer drug, therefore, the possibility of SJS/TEN like AE due to cutaneous toxicity should be excluded. Enfortumab vedotin (EV) is a humanized monoclonal antibody‐drug conjugate that delivers a microtubule‐disrupting agent, monomethyl auristatin E to cells that express nectin‐4, resulting in apoptotic death of tumoral cells [12]. Epidermal keratinocytes and skin appendages express nectin‐4. Then there is the potential for severe and possibly fatal cutaneous adverse reactions [12]. Grade 3–4 eruptions were reported in 13% of patients, most often after the first cycle [12]. Pruritic macules and extensive mucosal lesions were absent in contrast to bona fide SJS/TEN [12]. Histopathologically, inflammatory cells were sparse and a striking feature was the presence of abnormal mitotic figures [12]. Features described above were not observed in our case. Moreover, the result of DLST might support bona fide SJS in this case and it was caused by T cell‐mediated allergic reactions. The cutaneous toxicity by selpercatinib might be unlikely.

To date, SJS/TEN has been associated with various TKIs, including EGFR inhibitors, BRAF inhibitors, bcr‐abl/c‐KIT/PDGFR inhibitors, anaplastic lymphoma kinase (ALK) inhibitors, and multitargeted tyrosine kinase inhibitors (MKIs) [8]. EGFR inhibitors were the most frequent cause of SJS/TEN among those various TKIs [13]. Skin detachment observed in SJS/TEN is caused by apoptotic cell death of keratinocytes [7]. There are three distinct mechanisms of apoptosis: the Fas–Fas ligand pathway; the perforin/granzyme system; and granulysin [7]. In EGFR inhibitor associated SJS/TEN, release of granulysin by cytotoxic T cells was the cause of the apoptosis of the keratinocytes [13]. The prodromal phase of SJS/TEN by EGFR inhibitors ranges from 5 to 64 days [13]. Anti‐PD1 immunotherapy therapy “primes” the immune system then later on severe reactions may occur in BRAF inhibitors [14]. To the best of our knowledge, there have been no prior reports of SJS/TEN caused by selpercatinib, possibly because that is a relatively new drug, although mechanistic insights causing SJS/TEN by selpercatinib has not been unveiled.

In conclusion, we report a case of SJS induced by selpercatinib. This is the first case report of selpercatinib‐induced SJS. This case suggests that endocrine surgeons, oncologists, and dermatologists should be aware of the possibility of severe drug eruptions induced by selpercatinib.

## Conflicts of Interest

Author H.S. is an editorial board member of The Journal of Dermatology. To minimize bias, Authors H.S. was excluded from all editorial decision‐making related to the acceptance of this article for publication.
